# *Myrcia paivae* O.Berg (*Myrtaceae*) Essential Oil, First Study of the Chemical Composition and Antioxidant Potential

**DOI:** 10.3390/molecules27175460

**Published:** 2022-08-25

**Authors:** Ângelo Antônio Barbosa de Moraes, Celeste de Jesus Pereira Franco, Oberdan Oliveira Ferreira, Everton Luiz Pompeu Varela, Lidiane Diniz do Nascimento, Márcia Moraes Cascaes, Dehon Ricardo Pereira da Silva, Sandro Percário, Mozaniel Santana de Oliveira, Eloisa Helena de Aguiar Andrade

**Affiliations:** 1 Faculdade de Engenharia Química, Universidade Federal do Pará, Rua Augusto Corrêa S/N, Guamá, Belém 66075-900, Pará, Brazil; 2 Laboratório Adolpho Ducke—Coordenação de Botânica, Museu Paraense Emílio Goeldi, Av. Perimetral, 1901, Terra Firme, Belém 66077-830, Pará, Brazil; 3 Programa de Pós-Graduação em Biodiversidade e Biotecnologia—Rede Bionorte, Instituto de Ciências Biológicas, Universidade Federal do Pará, Rua Augusto Corrêa S/N, Guamá, Belém 66075-900, Pará, Brazil; 4 Laboratório de Pesquisas em Estresse Oxidativo, Instituto de Ciências Biológicas, Universidade Federal do Pará, Rua Augusto Corrêa S/N, Guamá, Belém 66075-900, Pará, Brazil; 5 Programa de Pós-Graduação em Química, Universidade Federal do Pará, Rua Augusto Corrêa S/N, Guamá, Belém 66075-900, Pará, Brazil

**Keywords:** Amazonia, natural products, phytochemicals, terpenes

## Abstract

The Myrtaceae family is one of the most representative in the Amazon. Several species have high added-value pharmacological potential. In order to contribute to the knowledge of the aromatic profile of Myrtaceae species from the Amazon, the present study presents the first report on the productivity, chemical composition, and antioxidant profile of the essential oil (EO) of *Myrcia paivae*. Dry leaves of the species were submitted to hydrodistillation to obtain their EO. The EO performance was calculated on a moisture-free basis and the analysis of the chemical profile was carried out by GC/MS. The determination of the antioxidant capacity was assessed by means of the antioxidant capacity equivalent to the inhibition Trolox of the ABTS^•+^ and DPPH^•^ radicals. The results indicate that EO performance was equivalent to 1.69%. As for the chemical composition, hydrocarbon monoterpenes were predominant in the sample (>77%); terpinolene (14.70%), α-phellandrene (14.69%), γ-terpinene (9.64%), sylvestrene (7.62%), α-thujene (6.46%), and α-pinene (6.39%) were the constituents with higher content. Regarding the antioxidant capacity, the results show that the EO presented good results in the inhibition of ABTS^•+^ (0.886 ± 0.226 mM L^−1^) and DPPH^•^ (2.90 ± 0.083 mM L^−1^), which can be attributed to the high monoterpene content in the sample.

## 1. Introduction

Essential oils are complex substances that are present in several aromatic plants. They are obtained from various parts of these plants, such as: leaves, flowers, fruits, seeds, buds, rhizomes, roots, and barks [1,2,3]. Essential oils play a key role in plant organisms, acting as antimicrobial agents [4,5,6].

Among the properties present in essential oils, the antioxidant potential is highlighted, with a focus on the search for natural antioxidants that aim to inhibit and reduce free radicals produced in excess by oxidative stress. These free radicals may be involved in triggering several pathophysiological processes, such as: cancer, Parkinson’s disease, Alzheimer’s disease, metabolic syndromes, and early aging [7]. Essential oils are made up of different organic compounds containing double combination carbon connections, in addition to hydroxyl groups, which can cause hydrogen release, thus inhibiting free radicals and minimizing the effects of oxidative stress [8].

Myrtaceae is a botanical family of species that produce essential oils, and is present in several Brazilian biomes, with the genus *Myrcia* being one of the main genera of this family. Its species that have antioxidant properties as observed in their essential oils include *Myrcia Sylvatica* [9], *M. splendens* [10], *M. palustres* [11], *M. oblongata* [12] and *M. tomentosa* [13]. However, some species of this genus are still unknown, both regarding the chemical composition of their essential oils and their antioxidant potential, including *Myrcia paivae*.

*M. paivae* is a species of tree that occurs in the Amazon biome in the North (Acre, Amazonas, Pará, Rondônia, Roraima) and Central-West (Mato Grosso) regions, in both solid ground and floodplain forests [14]. This species is considered a medicinal plant in the Amazon Region, and infusions of its leaves are used in the preparation of teas good for drinking during pregnancy and in the treatment of diabetes [15]. In view of the significant importance of this species, in this study, we evaluated for the first time the chemical composition and antioxidant profile of the essential oil of *M, paivae*.

## 2. Results

### 2.1. Yield of Essential Oil

The yield of the essential oil of *M. paivae* was 1.69%, higher than that found in the essential oil of some of the species in this genus, such as the essential oil of two specimens of *M. eximia* (A and B), which presented the respective yields 0.01% and 0.36% [16]. The difference in the yields of the essential oils of *Myrcia* was observed in several studies, such as in the essential oil of *M. sylvatica*, in which the levels varied from 0.9 to 1.7% [17]. In another study, the essential oils of *Myrcia bracteata*, *M. cuprea,* and *M. sylvatica* specimens showed outputs with a variation of 0.1 to 0.3% [18].

### 2.2. Chemical Composition

The chemical constituents found in the essential oil from the dry leaves of *M. paivae,* are listed in Table 1. In total, 74 constituents were identified and quantified using GC/MS.

The essential oil of *M. paivae* was characterized by hydrocarbon monoterpenes (77.93%), followed by hydrocarbon sesquiterpenes (10.16%) and oxygenated monoterpenes (7.97%). Studies carried out with the essential oils of other *Myrcia* species show the prevalence of monoterpenes and sesquiterpenes in their composition [13,16,21,22,23]. Terpinolene (14.70%), α-phellandrene (14.69%), *γ*-terpinene (9.64%), sylvestrene (7.62%), α-thujene (6.46%), and α-pinene (6.39%) were the majority constituents found in the essential oil of *M. paivae.*

Gatto et al. [23] found the majority constituents of the essential oil of *Myrcia hatschbachii* were (*E*)-calamenene (19.10%), (*E*)-caryophyllene (10.96%), and spathulenol (5.03%). α-pinene, one of the majority constituents of *M. paivae*, was found in low concentrations in the oil of *M. hatschbachii*. *γ*-Elemene (12.52%), germacrene D (11.45%), and *(E)-*caryophyllene (10.22%) were the majority compounds found in a sample of the essential oil of *M. tomentsa* [13]. The authors also point out that in another sample of the same species, there was a prevalence of spathulenol (40.70%). Ferreira et al. [22] studied three *M. multiflora* specimens and obtained *α*-bulnesene (26.79%) and pogostol (21.27%) as majority constituents in specimen A, and (*E*)-nerolidol in specimens B and C, with the content of 44.4% and 92.21%, respectively. Ferreira et al. [16] found the prevalence of (*E*)-caryophyllene in the specimens of *M. eximia* (15.71% and 20.0%). α-Guaiene (25.89%) and α-bulnesene (13.39%) were the main compounds in the essential oil of *M. palustris* (Santos et al. 2021). These results point out that essential oils of *Myrcia* species have a great chemical variability depending on the species studied. In addition, other factors can contribute to the differences in chemical composition, such as geographic location and seasonal and circadian factors [2].

According to Andrade-Ochoa et al. and Diniz do Nascimento et al. [24,25], the biological properties of essential oils may be directly related to the presence of the majority constituents and/or the synergic and antagonistic effects performed by all volatile components present in the samples. Terpinolene is a monoterpene hydrocarbon that has several biological properties reported in the literature.

According to Menezes et al. [26], essential oils containing this monoterpene in high concentrations have larvicidal, insecticidal, antifungal, antibacterial, antiproliferative, cytoprotective, antiviral, and antimicrobial properties. Pavela [27] points out that this compound has larvicidal activity against the larvae of the mosquito *Culex quinquefasciatu.* Aydin et al. [28], state that terpinolene is a powerful antiproliferative agent of tumor cells and a potential candidate for use as an anticancer.

With regard to α-phellandrene, Zhang et al. [29] point out that this volatile component has antifungal activity against *Penicillium cyclopium*. Lima et al. [30] point out that the compound also showed antinociceptive activity in rodents, acting in the glutamatergic, opioid, nitrergic, cholinergic, and adrenergic systems. İşcan et al. [31], show evidence that α-phellandrene derivatives, converted by microbial biotransformation, show antibacterial activity against different types of bacteria, and antifungal activities in relation to types of the *Candida* genus.

Gong and Ren [32] show that γ-terpinene induced the mortality of 86.7 ± 2.9% of the larvae of the caterpillar *Helicoverpa armigera* at a concentration of 250 μg mL^−1^, with an average inhibitory concentration (CL_50_) equivalent to 150.15 μg mL^−1^. Waller et al. [33] and Tahvilian et al. [34] state that essential oils containing γ-terpinene presented potential antifungal activity in relation to fungi species of *Candida, Aspergillus, and Cryptococcus*. 

According to Guerra-Boone et al. [35], sylvestrene is the second-largest constituent of the essential oil of *Schinus molle* leaves (22.3–34.3%). In accordance with the authors, the essential oil of this species showed antibacterial activity against *Staphylococcus aureus* and *Streptococcus pyogenes*. Joshi et al. [36] claim that the essential oil of *Senecio graciliflorus* presents α-pinene (15.0%) and α-thujene (10.0%) as its main components. As stated by the authors, this oil significantly inhibited the growth of the bacteria *Pseudomonas aeruginosa*, *Escherichia coli*, *Staphylococcus aureus,* and *Salmonella typhi*.

According to Karapandzova et al. [37], the essential oils of the berries and leaves of *Juniperus excelsa* presented α-pinene as a majority component, with levels of 70.81% and 33.83% respectively. In accordance with the authors, the berries oil showed the best result for maximum inhibition of the concentration of the *Haemophilus influenzae* bacteria (MIC = 31 μL mL^−1^). Furthermore, the authors state that the activity of the leaf oil was lower for all the bacteria analyzed, with MIC equivalent to 125 μL mL^−1^.

α-Pinene is a well-represented compound in several essential oils, which has antibiotic, antimicrobial, anticonvulsant, anticoagulant, antitumoral, anti-inflammatory, and antimalaria properties, among others [38]. Moreover, Aydin et al. [28] point out that α-pinene presents anticancer and genotoxic properties in cell lines of neuroblastoma in mice.

### 2.3. Antioxidant Profile

**Figure 1:**
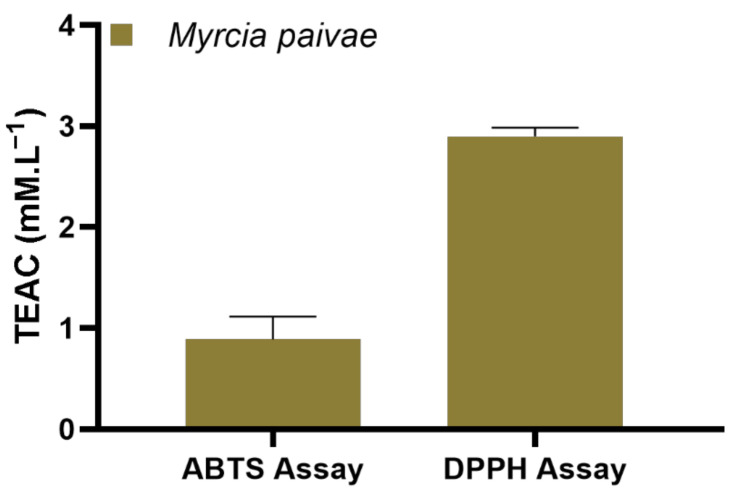
Trolox Equivalent Antioxidant Capacity (TEAC) of the essential oil of *Myrcia paivae*. Values are expressed as mean and standard deviation (*n* = 3) of TEAC. Student’s t-test was used to compare OE of *Myrcia paivae* to the Trolox standard (1 mM L^−1^).

We used 2,2’-Azino-bis (3-ethylbenzothiazoline-6-sulfonic acid (ABTS^•+^) and 2,2-diphenyl-1-picrylhydrazyl (DPPH^•^) radicals to evaluate the antioxidant capacity of the essential oil of *M. paivae* (Figure 1). According to the results, the Trolox equivalent antioxidant capacity (TEAC) required to eliminate the ABTS^•+^ and DPPH^•^ radicals of the sample was 0.88 mM L^−1^ and 2.90 mM L^−1^, respectively. As for the TEAC, the essential oil showed inhibition of the ABTS^•+^ radical similar (*p* = 0.557) to the standard (1 mM.L^−1^ of Trolox). On the other hand, the essential oil inhibited the DPPH^•^ radical in a significant manner (*p* = 0.001) in comparison with the standard. Such results show that there was a difference in antioxidant capacity, and this difference was also observed in the study carried out by [4], in the essential oil of two specimens of *Eugenia Florida* collected in Magalhães Barata, Pará, Brazil.

Within the *Myrcia* genus, there are studies that demonstrate the antioxidant potential present in the essential oils of some species, such as: *Myrcia hatschbachii* [23], *M. sylvatica* [9], *M. oblongata* [12], *M. splendens* [10], and *M. palustris* [11].

The antioxidant potential of the essential oil of *M. Paivae* in both the inhibition of the ABTS^•+^ radical and the inhibition of the DPPH^•^ radical was higher compared to the essential oil of two specimens of *Myrcia tomentosa* (A and B) collected in Magalhães Barata, Pará, Brazil. The essential oil of *M. tomentosa* (A) showed an antioxidant capacity of 53.6 ± 0.15 mM L^−1^ in the ABTS^•+^ radical, and 213.0 ± 0.90 mM L^−1^ in DPPH^•^. In turn, specimen B of *M. tomentosa* presented 0.333 ± 0.24 mM L^−1^ in the ABTS^•+^ assay and 208.5 ± 0.94 mM L^−1^ in the DPPH^•^ assay [13]. The difference in antioxidant capacity may be related to the chemical profile of each essential oil, as the essential oils of *Myrcia tomentosa* (A and B) were strongly characterized by hydrocarbons and oxygenated sesquiterpenes [13], while the essential oil of *M. paivae* was characterized by hydrocarbon monoterpenes. The significant monoterpene content in this essential oil may have corroborated the antioxidant potential of the sample studied, since the majority of terpinolene, α-phellandrene, and γ-terpinene are described in the literature as excellent antioxidant compounds [28,39,40]. 

## 3. Materials and Methods

### 3.1. Botanical Material

*M. paivae* aerial parts were collected in the coastal region of the State of Pará, in the city of Peixe-Boi, Brazil, whose geographic coordinates are 1°06′28.4″ S 47°20′13.1″ W. The sample was collected on 26 November 2019, and the exsiccate was incorporated into the collection of herbaria João Murça Pires (MG) of the Museu Paraense Emílio Goeldi, in the collection of Aromatic Plants of the Amazon, Belém, Pará, Brazil, and received the registration of MG243647.

The *M. paivae* leaves were dried in a convection oven at 35 °C for 5 days. Then, the material was ground and the moisture content was quantified using an infrared moisture detector at 115 °C for 30 min.

### 3.2. Extraction of Essential Oils

The samples were subjected to hydrodistillation in modified Clevenger-type glass systems for 3 h, coupled with a refrigeration system to maintain the water condensation at around 12 °C. After extraction, the oils were centrifuged for 5 min at 3000 rpm, dehydrated with anhydrous sodium sulfate, and centrifuged again under the same conditions. Oil yield was calculated in mL/100 g. The oils were stored in amber glass ampoules, sealed with flame, and stored in a refrigerator at 5 °C.

### 3.3. Chemical Composition Analysis

The chemical composition of the EO of *M. Paivae* was analyzed using a Shimadzu QP-2010 plus (Kyoto, Japan), a gas chromatography system equipped with a capillary column (30 m × 0.25 mm; 0.25 µm film thickness) coupled with a mass spectrometer (GC/MS) (Shimadzu, Kyoto, Japan). The programed temperature was maintained at 60–240 °C at a rate of 3 °C/min, with an injector temperature of 250 °C, helium as the carrier gas (linear velocity of 32 cm/s, measured at 100 °C), and a splitless injection (1 μL of a 2:10 hexane solution), using the same operating conditions as described in the literature [3,4,5]. The components were quantified using gas chromatography (GC) on a Shimadzu QP-2010 system (Kyoto, Japan), equipped with a flame ionization detector (FID) (Kyoto, Japan), under the same operating conditions as before, except the carrier was hydrogen gas. The retention index for all volatile constituents was calculated using a homologous series of *n*-alkanes (C_8_–C_40_) Sigma-Aldrich (St. Louis, MO, USA), in accordance with Van den Dool and Kratz [41]. The components were identified by comparison (i) of the experimental mass spectra with those compiled in libraries (reference) and (ii) their retention indices to those found in the literature [19,20].

### 3.4. Trolox Equivalent Antioxidant Capacity

The ABTS^•+^ and DPPH^•^ assay methods were used for the assessment of the TEAC of essential oils. The antioxidant potential of the sample was determined according to their equivalence to the potent antioxidant, Trolox (6-hydroxy-2,5,7,8-tetramethylchromono-2-carboxylic acid; Sigma-Aldrich; 23881-3; São Paulo, Brazil), and a water-soluble synthetic vitamin E analog. All values found for the samples were compared to the Trolox standard (1 mM L^−1^).

#### 3.4.1. ABTS Assay

The ABTS assay was determined according to the methodology adapted from Miller et al. [42] and modified by Re et al. [43]. ABTS^•+^ 2.45 mM L^−1^ (Sigma-Aldrich; A1888; São Paulo, Brazil) was prepared using 7 mM.L^−1^ ABTS^•+^ and 140 mM L^−1^ of potassium persulfate (K_2_O_8_S_2_; Sigma Aldrich; 216224; São Paulo, Brazil) incubated at room temperature without light for 16 h. Then, the solution was diluted with phosphate-buffered saline until it reached an absorbance of 0.700 (± 0.02) at 734 nm.

To measure the antioxidant capacity, 2.97 mL of the ABTS^•+^ solution was transferred to the cuvette, and the absorbance at 734 nm was determined using a spectrophotometer 800 XI (Femto; São Paulo, Brazil). Then, 0.03 mL of the sample was added to the cuvette containing the ABTS^•+^ radical and, after 5 min, the second reading was performed. The synthetic antioxidant Trolox was used as a standard solution for the calibration curve (y = 0.4162x − 0.0023, where y represents the value of absorbance, and x the value of concentration, expressed as mM.L^−1^; R^2^ = 0.9789). The results were expressed as mM L^−1^. The values found for the samples were compared to the Trolox standard (1 mM L^−1^).

#### 3.4.2. DPPH Assay

The test was carried out according to the method proposed by [44]. To measure the antioxidant capacity, initially, the absorbance of DPPH^•^ solution (Sigma-Aldrich; D9132; São Paulo, Brazil) 0.1 mM L^−1^ diluted in ethanol was determined. Subsequently, 0.95 mL of DPPH^•^ solution and 0.05 mL of the sample were mixed and placed in a water bath at 30 °C for 30 min. Thereafter, the absorbances were determined in a spectrophotometer 800 XI (Femto; São Paulo, Brazil) at 517 nm. The synthetic antioxidant Trolox was used as a standard solution for the calibration curve (y = 0.1271x − 0.0023, where y represents the value of absorbance and x the value of concentration, expressed as mM L^−1^; R^2^ = 0.9856). The results were expressed as mM.L^−1^. The values found for the samples were compared to the Trolox standard (1 mM L^−1^). Further details of the antioxidant potential experiment can be found in the Supplementary Material S1.

## 4. Conclusions

*M. Paivae* is a species of *Myrtaceae* that occurs in the Amazon Region, whose chemical composition and antioxidant profile are presented for the first time in this work. The output of essential oil was equivalent to 1.69%. Regarding the chemical profile, monoterpenes hydrocarbons showed a higher content (77.93%), with a prevalence of terpinolene (14.70%), α-phellandrene (14.69%), and γ-terpinene (9.64%). Antioxidant profile tests showed good results for the essential oil of *M. paivae* when compared to the Trolox standard. The TEAC in the ABTS^•+^ assay resulted in 0.886 ± 0.226 mM L^−1^, while for the DPPH assay, the result was 2.90 ± 0.083 mM L^−1^. Regarding future perspectives, it would be interesting to deepen the study by carrying out a seasonal and circadian rhythm study for samples of *M. Paivae.*

## Figures and Tables

**Table 1:** Chemical composition of the essential oil extracted from the leaves of *Myrcia paivae* through hydrodistillation.

IR_C_	IR_L_	Constituents	Area (%)
921	924	*α*-Thujene	6.46
941	932	*α*-Pinene	6.39
968	969	Sabinene	0.13
971	974	*β*-Pinene	0.53
986	988	Myrcene	5.79
1006	1002	*α*-Phellandrene	14.69
1016	1014	*α*-Terpinene	5.21
1020	1022	*o*-Cymene	1.41
1027	1025	Sylvestrene	7.62
1029	1025	*β*-Phellandrene	4.25
1043	1044	(*E*)-*β*-Ocimene	0.74
1059	1054	*γ*-Terpinene	9.64
1092	1086	Terpinolene	14.70
1097	1095	Linalool	0.49
1108	1108	1,3,8-*p*-Menthatriene	0.13
1110	1114	*endo*-Fenchol	0.03
1117	1118	(*Z*)-*p*-Menth-2-en-1-ol	0.35
1124	1128	*allo*-Ocimene	0.24
1131	1135	2-vinyl-Anisole	0.13
1144	1140	*trans*-Verbenol	0.05
1163	1165	Borneol	0.34
1175	1174	Terpinen-4-ol	3.66
1180	1179	*p*-Cymen-8-ol	0.71
1188	1186	*α*-Terpineol	2.63
1191	1195	(*Z*)-Piperitol	0.11
1203	1207	(*E*)-Piperitol	0.13
1222	1227	Nerol	0.01
1242	1244	Carvotanacetone	0.02
1255	1257	Methyl citronellate	0.01
1295	1289	Thymol	0.15
1346	1345	*α*-Cubebene	0.05
1368	1373	*α*-Ylangene	0.06
1373	1374	*α*-Copaene	0.36
1387	1390	Sativene	0.02
1407	1409	α-Gurjunene	0.01
1420	1417	(*E*)-Caryophyllene	3.99
1427	1430	*β*-Copaene	0.41
1430	1434	*γ*-Elemene	0.08
1436	1439	Aromadendrene	0.13
1440	1442	6,9-Guaiadiene	0.02
1443	1448	(*Z*)-Muurola-3,5-diene	0.03
1447	1451	*trans*-Muurola-3,5-diene	0.10
1451	1452	*α*-Humulene	0.54
1458	1458	*allo*-Aromadendrene	0.05
1460	1461	(*Z*)-Cadina-1(6),4-diene	0.06
1470	1475	(*E*)-Cadina-1(6),4-diene	0.19
1473	1478	*γ*-Muurolene	0.26
1477	1483	*α*- Amorphene	0.12
1484	1489	*β*-Selinene	0.36
1489	1493	*trans*-Muurola-4(14),5-diene	0.08
1492	1495	*γ*-Amorphene	0.27
1493	1496	Viridiflorene	0.20
1497	1500	*α*-Muurolene	0.32
1504	1511	*δ*-Amorphene	0.29
1511	1513	*γ*-Cadinene	0.24
1515	1520	7-*epi*-*α*-Selinene	0.01
1521	1522	*δ*-Cadinene	1.03
1523	1528	Zonarene	0.21
1529	1533	(*E*)-Cadina-1,4-diene	0.07
1532	1540	Selina-4(15),7(11)-diene	0.06
1534	1537	*α*-Cadinene	0.06
1540	1544	*α*-Calacorene	0.36
1560	1564	*β*-Calacorene	0.12
1565	1565	(3*Z*)-Hexenyl benzoate	0.02
1574	1570	Dendrolasin	0.12
1581	1582	Caryophyllene oxide	0.46
1589	1592	Viridiflorol	0.16
1606	1608	Humulene epoxide II	0.01
1609	1618	1,10-di-*epi*-Cubenol	0.11
1625	1627	1-*epi*-Cubenol	0.35
1639	1640	epi-*α*-Muurolol	0.29
1643	1644	*α*-Muurolol	0.12
1648	1652	Himachalol	0.05
1651	1652	*α*-Cadinol	0.29
		Hydrocarbon monoterpenes	77.93
		Oxygenated monoterpenes	7.97
		Hydrocarbon sesquiterpenes	10.16
		Oxygenated sesquiterpenes	1.96
		Others	0.87
		Total	98.89

IR_C_: calculated from a series of n-alkanes (C_8_–C_40_) in a DB-5MS column capillary column, IR_L_: [19,20]; IR_C_: calculated retention index; IR_L_: literature retention index.

## Data Availability

Not applicable.

## Supplementary Materials

The following supporting information can be downloaded at: https://www.mdpi.com/article/10.3390/molecules27175460/s1, Supplementary Material S1: Further details of the antioxidant potential.

## Sample Availability

Samples of the compounds *Myrcia paivae* O.Berg (*Myrtaceae*) essential oil are available from the authors.
