# Supplementary material for: Myrcia paivae O.Berg (Myrtaceae) Essential Oil, First Study of the Chemical Composition and Antioxidant Potential
Source: Molecules. 2022 Aug 25;27(17):5460. doi: 10.3390/molecules27175460 (PMC9458249; doi:10.3390/molecules27175460)
Supplement: Supplementary file 1 [file molecules-27-05460-s001.zip › molecules-1871441-supplementary.pdf]

Myrcia paivae O.Berg (*Myrtaceae*) Essential Oil, First Study of the Chemical Composition and Antioxidant Potential

Supplementary Material S1

ABTS Assay

PREPARATION OF PBS pH 7.2 SOLUTION
Isotonic saline solution with pH 7.2 was used as a solvent in the preparation of the reagents. To prepare the phosphate-buffered saline solution (PBS), 1.48g of Na_2HPO_4 (dibasic sodium phosphate), 0.43 g of NaH_2PO_4 (monobasic sodium phosphate), and 7 g of NaCl (sodium chloride) were weighed. Then, they were dissolved in 1 L of distilled water and the pH of the solution was adjusted in the pH meter to 7.2.

PREPARATION OF THE ABTS$^{•+}$ STOCK SOLUTION
Diammonium salt of 2,2'-azinobis(3-ethylbenzothiazoline-6-sulfonic acid) – ABTS. ABTS$^{•+}$ stock solution was prepared 16 h prior to dosing. A solution of ABTS diammonium salt at 7 mM.L^{-1} was mixed with a solution of potassium persulfate ($K_2S_2O_8$) whose final concentration was 2.45 mM.L^{-1}.
(A) ABTS 7 mM.L^{-1}: 0.0768 g----20mL of PBS
(B) $K_2S_2O_8$ 140 mM.L^{-1}: 0.7560 g-----20mL of PBS
Note: 352 µL of the solution (A) was removed and discarded. After that, solution (A) was mixed with 352 µL of solution (B). Thus, 20 mL of ABTS$^{•+}$ stock solution was prepared. The final concentration of (A) in this mixture was 2.45 mM.L^{-1}.

PREPARATION OF THE ABTS$^{•+}$ WORKING SOLUTION
To prepare the working solution, ABTS$^{•+}$ stock solution was mixed with 200 mL of PBS in an Erlenmeyer flask until the absorbance at 734 nm was 0.700 ± 0.02. In this step, 2200 µL of ABTS$^{•+}$ stock solution was used, and added to PBS, then homogenized, and the spectrum was read.
- Calibrate spectrophotometer at 734 nm reading, zeroing with PBS.

PROCEDURE
Initially, 2970 µL of the ABTS$^{•+}$ working solution was placed in the cuvette, followed by the first reading (T0). Then, 30 µL of the sample or standard was transferred to the cuvette containing the ABTS$^{•+}$ working solution and, after 5 min, the second reading (T5) was performed. The reaction was measured in a spectrophotometer (Femto 800XI; São Paulo/SP) at 734 nm. Trolox (2.5 mM.L^{-1}) was used as a standard to obtain the standard curve.

TROLOX STANDARD CURVE

The TEAC standard curve was achieved by successively diluting the trolox standard stock solution (2.5 $mM.L^{-1}$), as described in Table S1. The curve was performed in triplicate, and at the end, the average absorbance of the values found was calculated.

Table S1. Trolox standard solution dilution protocol for standard curve.

Test tube	Trolox Standard (μL)	PBS (μL)	Final Trolox concentration ($mM.L^{-1}$)
A	100	0	2.5
B	80	20	2.0
C	60	40	1.5
D	40	60	1.0
E	20	80	0.5
F	0	100	0

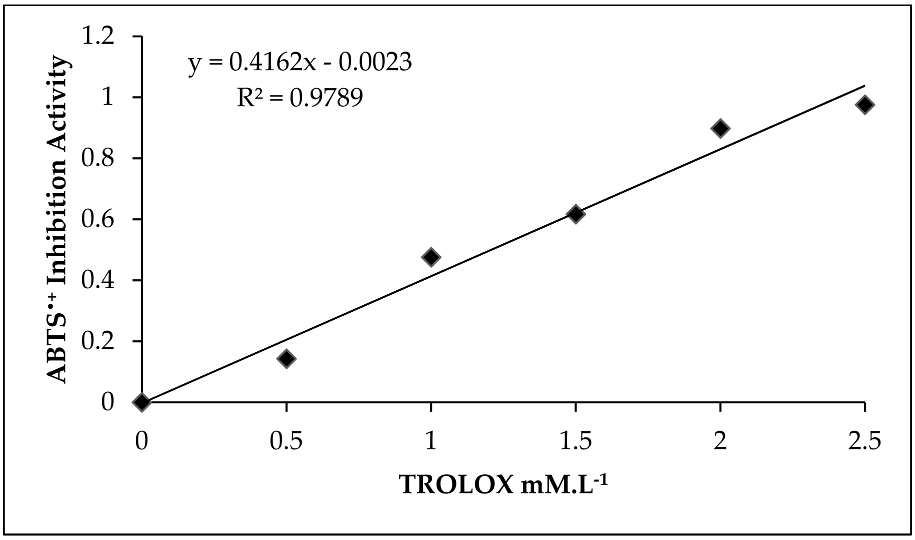

Based on the data obtained, the equation of the line was calculated, using the equation of least squares, obtaining the following equation of the line:

$$TEAC(mM.L-1) = \left(Abs_{sample} \pm \frac{b}{a}\right) \qquad Eq.(1)$$

Being:

Abs_{sample} = sample absorbance;

a = slope coefficient obtained for the calibration curve;

b = linear coefficient obtained for the calibration curve.

From the equation of the straight line, the final TEAC value of each sample was found, which was expressed in millimolar ($mM.L^{-1}$).

CALCULATION OF RESULTS

The total antioxidant activity (TAA) of each sample was calculated using the following formula:

$$TAA = \frac{(T_0 - T_5)}{T_0} \qquad Eq.(2)$$

Afterwards, the corrected value of the total antioxidant activity (TAAc) of each sample was calculated by subtracting the TAA value of the samples (essential oil) and the standard TAA value found for the Blank:

$$TAAc = (TAA - TAA_{Blank}) \qquad Eq.(3)$$

Subsequently, the value of the inhibition capacity was calculated through the equation of the straight line found from the realization of the Trolox standard curve (2.5 $mM.L^{-1}$).

$$TEAC\ (mM.L-1) = \frac{(TAAc + 0.0023)}{0.4162} \qquad Eq. (4)$$

DPPH Assay

PREPARATION OF THE WORKING SOLUTION OF THE DPPH•

Initially, 0.5 L of the DPPH• working solution was prepared. For this, 0.0197 g of DPPH (0.1 mM.L^{-1}) was weighed on an analytical balance and mixed with ethanol (0.5 L). Then, the initial absorbance reading of the DPPH• working solution (0.1 mM.L^{-1} in ethanol) was performed. Subsequently, 600 μL of DPPH solution, 350 μL of distilled water, and 50 μL of sample (essential oil) or standard were mixed and placed in a water bath at 37 °C for 30 min. After this period, the reading was performed in a spectrophotometer (Femto 800XI; São Paulo/SP) at 517 nm. Trolox (1 mM.L^{-1}) was used as the standard for the calibration curves.

Trolox standard curve

The TEAC standard curve was performed by successively diluting the Trolox standard solution (1 mM.L^{-1}), as described in Table S2. The curve was performed in triplicate and, at the end, the average absorbance of the values found was calculated.

Table S2. Trolox standard solution dilution protocol for standard curve.

Test tube	Trolox Standard (μL)	PBS (μL)	Final Trolox concentration (mM.L^{-1})
A	200	0	1
B	160	40	0.8
C	120	60	0.6
D	80	120	0.4
E	40	160	0.2
F	0	200	0

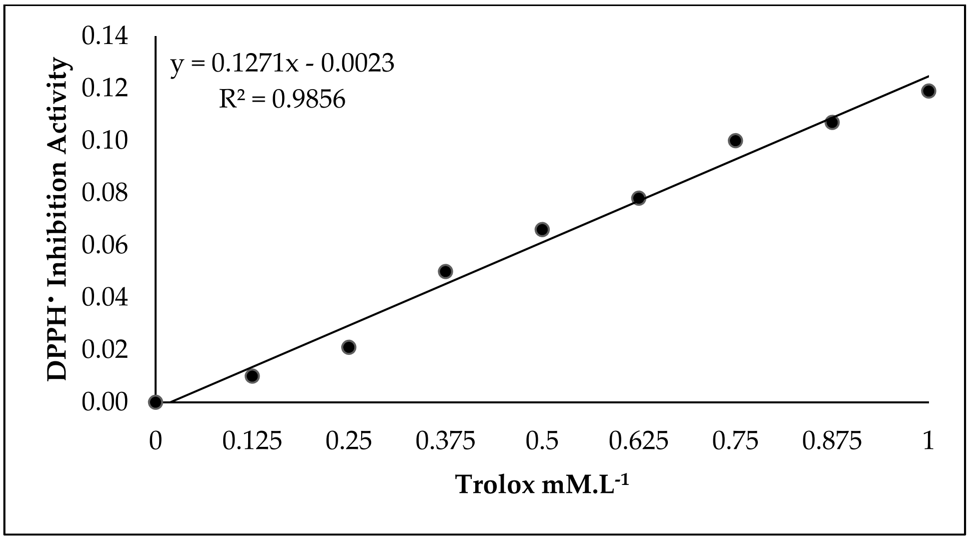

Based on the data obtained, the equation of the line was calculated, using the equation of least squares, obtaining the following equation of the line:

$$TEAC\ (mM.L-1) = \left(\frac{Ab_{sample} \pm b}{a}\right) \qquad Eq. (6)$$

which was:
Abs_{sample} = sample absorbance;
a = slope obtained for the calibration curve;
b = linear coefficient obtained for the calibration curve.

CALCULATION OF RESULTS

The absorbance values obtained were subtracted from the initial absorbance of the DPPH• working solution and from the absorbance of the blank.

$$\left(Abs_{DPPH\cdot} - Abs_{sample}\right) - \left(Abs_{DPPH\cdot} - Abs_{blank}\right) \qquad Eq. (7)$$

Being:
$Abs_{DPPH\bullet}$ = initial absorbance value of the DPPH• working solution
Abs_{sample} = sample reading absorbance value
Abs_{blank} = absorbance value of blank reading

After this calculation, the TEAC value of each sample was obtained from the equation of the straight line obtained through the standard curve and was expressed in mM.L^{-1}.

$$TEAC\ (mM.L - 1 = \left(\frac{Ab_{sample} + 0.0023}{0.1271}\right) \qquad Eq. (8)$$

The values of the antioxidant capacity of essential oil from *Myrcia paivae* leaves were compared to Trolox (1 mM.L^{-1}).

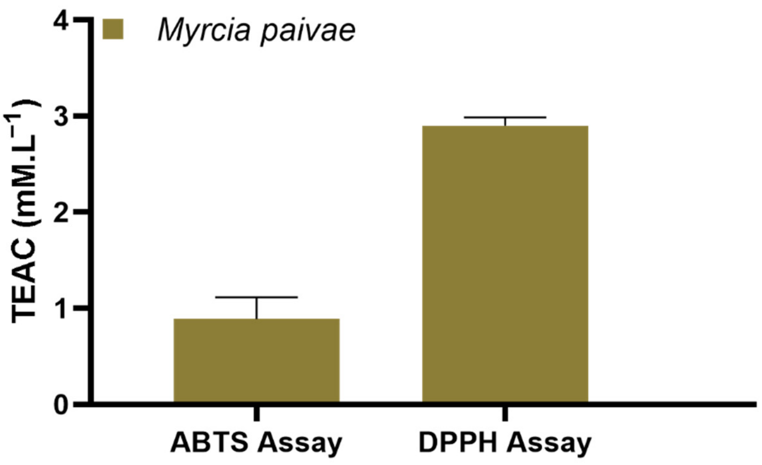

Figure S1. Trolox Equivalent Antioxidant Capacity (TEAC) of the essential oil of *Myrcia paivae*. Values are expressed as mean and standard deviation (n = 3) of TEAC. Student's t-test was used to compare OE of *Myrcia paivae* to the Trolox standard (1 mM.L^{-1}).
